# Bipolar radiofrequency catheter ablation between the left ventricular endocardium and great cardiac vein for refractory ventricular premature complexes originating from the left ventricular summit

**DOI:** 10.1002/joa3.12312

**Published:** 2020-02-14

**Authors:** Sayuri Tokioka, Seiji Fukamizu, Iwanari Kawamura, Takeshi Kitamura, Rintaro Hojo

**Affiliations:** ^1^ Department of Cardiology Tokyo Metropolitan Hiroo Hospital Tokyo Japan

**Keywords:** coronary sinus, dilated cardiomyopathy, electric impedance, radiofrequency catheter ablation, ventricular premature complexes

## Abstract

Ablation for ventricular arrhythmias originating from the left ventricular (LV) summit is sometimes challenging. Bipolar radiofrequency catheter ablation (RFCA) is effective for refractory arrhythmias; little is known about bipolar RFCA from the coronary venous system and the appropriate settings. We experienced three cases of ventricular premature complexes (VPCs) originating from the LV summit successfully treated by bipolar RFCA between the LV endocardium (irrigated catheters as active electrodes) and coronary venous system (8‐mm‐tip catheters as return electrodes). These cases showed that bipolar RFCA was effective for the VPCs originating from the LV summit; 8‐mm‐tip catheters were useful as return electrodes.

## Graphical abstract

We described 3 cases of VPCs originating from the LV summit successfully treated by bipolar RFCAs between the LV endocardium (irrigated catheters as active electrodes) and coronary venous system (8‐mm‐tip catheters as return electrodes). These cases showed that bipolar RFCA was effective for VPCs originating from the LV summit; an 8‐mm‐tip catheter was useful as a return electrode.

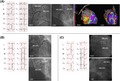

## 1 INTRODUCTION

Ablation for ventricular arrhythmias (VAs) originating from the left ventricular (LV) summit is sometimes challenging because of intramural origins.^1^, ^2^ Bipolar radiofrequency catheter ablation (RFCA) can reportedly create transmural lesions by using 2 ablation catheter tips as active electrodes (AEs) and return electrodes (REs).^3^ Previous reports have shown the effectiveness of bipolar RFCA for VAs originating from the LV summit.^4^ There are several bipolar RFCA settings and we have recently reported a case of successful bipolar ablation involving changing an irrigated catheter to an 8‐mm‐tip catheter to reduce the impedance.^5^ However, the effectiveness and safety of bipolar RFCA from the coronary venous system for VAs originating from the LV summit and the appropriate settings of bipolar RFCA remain unclear.

Here, we describe 3 cases of successful bipolar RFCA between the LV endocardium and great cardiac vein **(**GCV) for the treatment of the ventricular premature complexes (VPCs) originating from the LV summit.

## 2 METHOD

Bipolar RFCA was eligible for cases of VAs for which unipolar RFCA in the prior sessions failed or VA recurred, and the study was approved by the institutional ethics committee. Written informed consent was obtained from all patients. Electrophysiological studies (EPS) and catheter ablation were performed using a 3‐dimensional electro‐anatomical mapping system (Ensite; Abbott or CARTO; Biosense Webster). Bipolar RFCA between the LV endocardium and GCV was performed using irrigated catheters (TactiCath^TM^; Abbott or THERMOCOOL SMARTTOUCH® SF; Biosense Webster) in the LV endocardium as AEs, and 8‐mm‐tip catheters (Dual‐8^TM^ one Ablation Catheter; Abbott) in the GCV via the subclavian vein as REs with cardiac effusion monitoring by intracardiac echo (SOUNDSTAR; Biosense Webster). Impedance was carefully monitored during ablation, and ablation was stopped instantly when impedance increased or it decreased by more than 30 Ω.

## 3 CASE REPORT

We experienced 3 cases of VPCs originating from the LV summit successfully treated by bipolar RFCA (Table 1).

**Table 1:** Summary of catheter ablation

Case	#1	#2	#3
Age/Gender	61/M	58/M	60/M
Background	DCM	idiopathic	DCM
EF [%]	19.7	62.7	23.4
ECG	RBBB, inf. axis	LBBB, inf. axis	RBBB, inf. axis
AAD	bisoprolol	None	carvedilol, mexiletine
Unipolar RFCA			
Ablation site in the prior session	LV basal septum, epicardium	LCC, GCV, LV endo	GCV, LV endo, LV basal, epicardium
Ablation site in the same session as bipolar RFCA	GCV	None	None
Unipolar RFCA from GCV and/or LV endo			
Ablation site	GCV	GCV, LV endo	GCV, LV endo
Initial impedance [Ω]	150	140	150
Max output [W]	30 (irrigated catheter)	GCV: 25 LV endo: 45 (irrigated catheter)	GCV: 30 W, 50℃ LV endo: 50 W, 50℃ (nonirrigated catheter)
No. of unipolar RF	4	6	25
Total RF time [sec]	120	270	901
Bipolar RFCA between GCV and LV endo			
Distance [mm]	14.0	13.6	15.6
Average initial impedance [Ω]	151	186	176
Average impedance drop [Ω]	12	18	32
Max output [W]	35	34	40
No. of bipolar RF	4	7	2
Total RF time [s]	160	280	66

Abbreviations: AAD, anti‐arrhythmic drug; DCM, dilated cardiomyopathy; ECG, electrocardiogram; EF, ejection fraction; GCV, great cardiac vein; inf., inferior; LBBB, left bundle branch block; LCC, left coronary cusp; LV endo, opposite side of GCV in the LV endocardium; LV, left ventricular; M, male; RBBB, right bundle branch block; RF, radiofrequency; RFCA, radiofrequency catheter ablation.

### 3.1 Case 1

**Figure 1:**
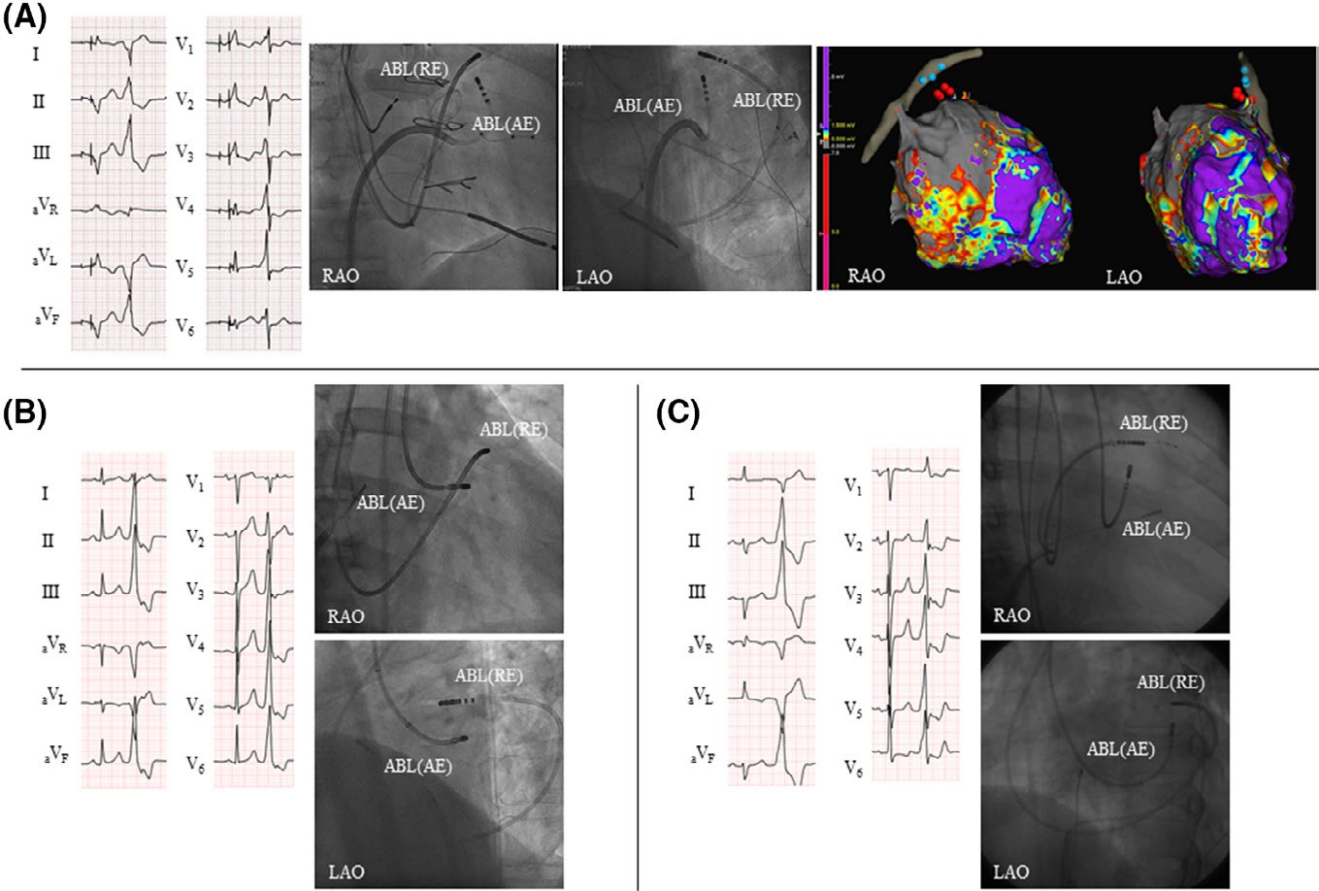
12‐lead electrocardiograms (ECGs) and fluoroscopic views for the 3 cases. A, Case 1. The 12‐lead ECG shows biventricular pacing (the first beat) and ventricular premature complex (VPC, the second beat) with the right bundle branch block and inferior axis. Fluoroscopic views show the catheters during bipolar ablation between the left ventricular (LV) endocardium (active electrode (AE); irrigated catheter) and great cardiac vein (GCV, return electrode (RE); an 8‐mm‐tip catheter). EnSite images of the left ventricle and coronary sinus show the ablation sites. The red tags are bipolar ablation sites, and the blue tags are unipolar ablation sites from the GCV. B, Case 2. The 12‐lead ECG shows the sinus rhythm (the first beat) and VPC (the second beat) with the left bundle branch block and inferior axis. Fluoroscopic views show the catheters during bipolar ablation between the LV endocardium (AE, irrigated catheter) and anterior interventricular vein (GCV, RE, 8‐mm‐tip catheter). C, Case 3. The 12‐lead ECG shows the sinus rhythm (the first beat) and VPC (the second beat) with the right bundle branch block and inferior axis. Fluoroscopic views show the catheters during bipolar ablation between the LV endocardium (AE, irrigated catheter) and GCV (RE, 8‐mm‐tip catheter). ABL, ablation catheter; AE, active electrode; LAO, left anterior oblique; RAO, right anterior oblique; RE, return electrode

A 61‐year‐old man with dilated cardiomyopathy (DCM) and a history of ventricular tachycardia (VT) ablation for the ventricular septum and epicardium and double valve replacement (DVR) was admitted to our hospital for heart failure. He had a cardiac resynchronization therapy defibrillator generator, and his biventricular pacing rate decreased to 86% because of frequent VPCs (Figure 1A). The morphology of the VPCs was similar to that of VT, which had been treated in the previous session. In EPS, a good pace‐map was obtained for the GCV. A prepotential preceding the surface QRS at 54 ms and a QS pattern were recorded by the unipolar electrogram in the GCV when the VPCs were present. After unsuccessful unipolar RFCA from the GCV, bipolar RFCA performed between the LV endocardium (an irrigated catheter; AE) and GCV (an 8‐mm‐tip catheter; RE) abolished the VPC. After ablation, the VPCs decreased considerably, and the biventricular pacing rate increased to 95%; BNP was decreased to 85.7 pg/mL from 368.9.

### 3.2 Case 2

A 58‐year‐old man who previously underwent unipolar RFCA for VPCs originating from the LVOT experienced recurrent VPCs (Figure 1B). He had severe palpitation and a Holter electrocardiogram (ECG) recorded 36 205 VPCs a day. In EPS, a good pace‐map was obtained in the GCV. Adequate unipolar RFCA was performed in the left coronary cusp, GCV and LV endocardium in the prior session. In this session, bipolar RFCA between the LV endocardium (an irrigated catheter; AE) and GCV (an 8‐mm‐tip catheter; RE) was performed, and the VPCs were abolished. After the procedure, the number of VPCs decreased to 864 a day in the Holter ECG.

### 3.3 Case 3

A 60‐year‐old man with DCM was admitted to our hospital owing to frequent VPCs (Figure 1C). The Holter ECG recorded 28 967 VPCs a day under antiarrhythmic drugs and the ejection fraction (EF) decreased to 23.4%. He underwent 2 sessions of unipolar RFCA for VPCs originating from the LV summit. In the previous sessions, unipolar RFCA from the GCV and LV epicardium was unsuccessful. In this session, a good pace‐map was observed in the GCV, and a prepotential preceding the surface QRS at 35 ms and a QS pattern were recorded by the unipolar electrogram during VPCs. Bipolar RFCA between the LV endocardium (an irrigated catheter; AE) and GCV (an 8‐mm‐tip catheter; RE) abolished the VPCs. After ablation, 5887 VPCs other than the target VPC remained in the Holter ECG, and the EF was improved to 32.0%.

## 4 DISCUSSION

In this case series, we identified two important clinical issues. First, bipolar RFCA between the LV endocardium and GCV was effective for refractory VPCs originating from the LV summit. In general, unipolar RFCA from the GCV and anterior intraventricular vein was attempted for VAs originating from the LV summit; however, it is sometimes unsuccessful because of intramural origin of the VAs.^1^ There are some reports on bipolar ablation for VAs originating from the LV summit;^4^ however, the methods are not established. This report showed the effectiveness of bipolar ablation from the coronary venous system. Second, an 8‐mm‐tip catheter was shown to be useful as an RE in the GCV. There are various bipolar RFCA settings.^3^ In the cases presented in this report, an 8‐mm‐tip catheter was used as an RE to avoid high impedance, whereas an irrigated catheter was used as an AE in the LV endocardium to reduce the risk of thrombosis in the LV. RFCA from the GCV was sometimes discontinued because of high impedance. Moreover, higher impedance is expected in bipolar RFCA than in unipolar RFCA. A previous study reported that bipolar RFCA using an 8‐mm‐tip catheter as RE recorded significant lower impedance and lower temperature compared with a 4‐mm‐tip catheter.^6^ This observation was consistent with the previous ex vivo study.^3^ This may be related to the current density; the size of the 2 catheters and the distance between them may regulate current density, which produces lesions. In these cases, bipolar RFCA was performed without high impedance. An 8‐mm‐tip catheter can be suitable for use as an RE in the GCV.

The appropriate setting remains unclear because of the limited number of patients. Safety is one of the most important issues, and reports on bipolar RFCA are limited. In addition, impedance and temperature can be monitored only by AEs while they cannot be monitored by REs in the current setting. Futyma P, et al reported the setup of bipolar RFCA which allows temperature monitoring from AEs and REs simultaneously using two RF generators.^6^ Technical advancement and further studies are needed to achieve better outcomes and to ensure safety of the procedure.

## 5 CONCLUSION

Bipolar RFCA between the LV endocardium and GCV was effective for the VPCs originating from the LV summit. An 8‐mm‐tip catheter may be useful as an RE in the GCV to reduce impedance.

## CONFLICTS OF INTERESTS

Authors declare no conflict of interests for this article.
